# Complete Genome Sequence of a *Carnation Mottle Virus* Infecting Hop Plants

**DOI:** 10.1128/genomeA.00416-15

**Published:** 2015-05-14

**Authors:** Yeonhwa Jo, Hoseong Choi, Won Kyong Cho

**Affiliations:** Department of Agricultural Biotechnology, College of Agriculture and Life Sciences, Seoul National University, Seoul, Republic of Korea

## Abstract

The *Carnation mottle virus* (CarMV) is a single positive-strand RNA virus belonging to the genus *Carmovirus*. The major natural host for CarMV is the carnation. In this study, using transcriptome data, we provide for the first time a nearly complete genome sequence of CarMV infecting hop plants.

## GENOME ANNOUNCEMENT

The *Carnation mottle virus* (CarMV) is a member of the genus *Carmovirus* in the family *Tombusviridae*. The genome of CarMV is composed of a single positive-strand RNA virus (1). The major natural hosts for CarMV are carnations (*Dianthus caryophyllus*) and pinks (*D. barbatus*), and infection of CarMV in a wide range of plants (e.g., *Zantedeschia* spp. [Calla lily], *Begonia elatior*, lettuce, and *Daphne × burkwoodii*) has been reported (2). CarMV is mechanically transmitted by sap in various experimental host species, including *Chenopodium* species (3). CarMV-infected carnations display mild mottling in young leaves and faint chlorosis in mature leaves; however, infection of CarMV does not always induce apparent viral disease symptoms.

To obtain information on viruses infecting hop plants (*Humulus lupulus*), we screened several hop transcriptomes. Of them, one recent study provided the draft genome and transcriptome for two hop cultivars and a wild hop that were sequenced by Illumina HiSeq 1500 (4). The transcriptome was obtained by mixing different tissues, including the leaf, flower, lupulin gland, and stem of the *H. lupulus* var. *lupulus* cultivar Shinshu Wase. We downloaded all RNA-Seq raw data and performed *de novo* assembly for the hop transcriptome using the Trinity program (version 2.0.2) (5). All obtained transcripts were blasted against the National Center for Biotechnology Information viral reference sequence (6). Interestingly, the four hop transcripts derived from the hop cultivar Shinshu Wase displayed strong sequence identity to the CarMV reference sequence (NC_001265.2) (1). Out of the four transcripts, the hop transcript that was 3,966 nucleotides (nt) long was the closest to the complete genome of CarMV. During the sequence alignment to the other four known CarMV genomes, we found that 12 nt and 15 nt at the 5′ and 3′ untranslated regions (UTRs), respectively, were shorter than the other CarMV genomes. However, the 5′ and 3′ UTR sequences for known CarMV genomes were identical. After removing thiamine nucleotides at the 5′ UTR, we obtained a nearly complete CarMV genome sequence 3,938 nt in length and named it CarMV isolate Japan (KR002041). The CarMV isolate Japan contains six open reading frames (ORFs) named CarMVgp1–CarMVgp6. Both CarMVgp1 and CarMVgp2 contain RNA-dependent RNA polymerase (RdRp) domains and share the same start codon. However, CarMVgp2 is produced by a translational readthrough product due to the amber stop codon of CarMVgp1. CarMVgp3 encodes an additional RNA-directed RNA polymerase. Two ORFs referred to as CarMVgp4 and CarMVgp5 encode movement proteins, and CarMVgp6 is the capsid protein gene. Of the five identified CarMV isolates, the BLASTn results showed a 98% nt sequence identity to both the isolate from India (AJ811998.1) and the isolate from the United States (X02986.1) (7). In summary, our study revealed for the first time the infection of CarMV in hop plants and provided a nearly complete genome sequence of CarMV using hop transcriptome data.

### Nucleotide sequence accession number. 

The genome sequence of *Carnation mottle virus* isolate Japan has been submitted to GenBank under the accession number KR002041.
